# Molecular and Antigenic Characterization of Reassortant H3N2 Viruses from Turkeys with a Unique Constellation of Pandemic H1N1 Internal Genes

**DOI:** 10.1371/journal.pone.0032858

**Published:** 2012-03-21

**Authors:** Yohannes Berhane, Helen Kehler, Katherine Handel, Tamiko Hisanaga, Wanhong Xu, Davor Ojkic, John Pasick

**Affiliations:** 1 National Centre for Foreign Animal Disease, Canadian Food Inspection Agency, Winnipeg, Manitoba, Canada; 2 Animal Health Laboratory, University of Guelph, Guelph, Ontario, Canada; University of Hong Kong, Hong Kong

## Abstract

Triple reassortant (TR) H3N2 influenza viruses cause varying degrees of loss in egg production in breeder turkeys. In this study we characterized TR H3N2 viruses isolated from three breeder turkey farms diagnosed with a drop in egg production. The eight gene segments of the virus isolated from the first case submission (FAV-003) were all of TR H3N2 lineage. However, viruses from the two subsequent case submissions (FAV-009 and FAV-010) were unique reassortants with PB2, PA, nucleoprotein (NP) and matrix (M) gene segments from 2009 pandemic H1N1 and the remaining gene segments from TR H3N2. Phylogenetic analysis of the HA and NA genes placed the 3 virus isolates in 2 separate clades within cluster IV of TR H3N2 viruses. Birds from the latter two affected farms had been vaccinated with a H3N4 oil emulsion vaccine prior to the outbreak. The HAl subunit of the H3N4 vaccine strain had only a predicted amino acid identity of 79% with the isolate from FAV-003 and 80% for the isolates from FAV-009 and FAV-0010. By comparison, the predicted amino acid sequence identity between a prototype TR H3N2 cluster IV virus A/Sw/ON/33853/2005 and the three turkey isolates from this study was 95% while the identity between FAV-003 and FAV-009/10 isolates was 91%. When the previously identified antigenic sites A, B, C, D and E of HA1 were examined, isolates from FAV-003 and FAV-009/10 had a total of 19 and 16 amino acid substitutions respectively when compared with the H3N4 vaccine strain. These changes corresponded with the failure of the sera collected from turkeys that received this vaccine to neutralize any of the above three isolates *in vitro*.

## Introduction

Type A influenza viruses belong to the family *Orthomyxoviridae* and have a segmented genome composed of 8 single-stranded RNAs of negative sense [1]. Although the natural reservoir of influenza A viruses are wild aquatic and shore birds, these viruses have been isolated from humans and a wide variety of other animal species including domestic poultry, swine, horses, minks, whales, cats and dogs [2], [3]. Influenza A viruses are classified on the basis of their hemagglutinin (HA) and neuraminidase (NA) surface glycoproteins of which 16 and 9 subtypes respectively have been identified to date. The antigenic characteristics of these two surface glycoproteins are important in eliciting protective antibody responses by the host [1], [2].

Although the HA protein is an important target of the host immune response and subtype specific anti-HA antibodies usually provide protection against infection with viruses of same HA subtype [4], new antigenic variants that result from the accumulation of point mutations (antigenic drift) within antigenic sites, frequently emerge in response to host immune pressure. This results in the appearance of antigenic variants within the same subtype that are capable of evading the host's immune response [5], [6]. In addition, new antigenic variants can also emerge by reassortment when influenza A viruses with different HA and NA subtypes co-infect the same animal (antigenic shift). This process leads to the appearance of new subtypes with dramatic changes in antigenicity [5], [7].

As a result of antigenic shift or drift, influenza viruses with novel combinations of gene segments or point mutations have been isolated from various animal species [1], [5], [8], [9]. In April 2009 a novel H1N1 influenza virus reassortant that contained genes from North American and Eurasian influenza viruses began infecting people in Mexico. This new influenza virus quickly spread into the USA and Canada and subsequently worldwide [10]. Soon after the initial human reports, this pandemic H1N1 (pH1N1) virus was isolated from a swine herd in Alberta, Canada in May 2009 [9], [11]. The first report of pH1N1 virus infection of turkeys came from Chile in August 2009 [12] and later from Ontario, Canada in October 2009 [13]. Involvement of these livestock species further complicated public health and veterinary regulatory responses due to the unknown roles that pigs, poultry and other domestic animals might play in the evolution of this virus. Consequently, emergence of such novel viruses with unique gene constellations not only poses a threat to human health, but might also have implications for animal health and international trade.

Swine influenza viruses have received increased attention in recent years from both the veterinary and public health authorities because of pigs being viewed as potential “mixing vessels” for the generation of novel viruses. In North America, the viruses that have been responsible for outbreaks in swine since 1998 have changed dramatically from the viruses that were responsible for outbreaks in the previous 70 years [14]. Prior to 1998 swine influenza was almost exclusively caused by viruses of the classical swine H1N1 lineage (cH1N1) which was first identified in North America in 1930 [15]. This H1N1 virus is also related to the 1918 Spanish influenza virus.

This situation changed in 1998 when a severe outbreak of swine influenza occurred in North Carolina followed by additional outbreaks in Minnesota, Iowa and Texas [14], [16]. The viruses responsible for the Minnesota, Iowa and Texas outbreaks were triple reassortant (TR) H3N2 viruses which contained human (HA, NA, PB1), swine (NS, NP, M) and avian (PB2, PA) influenza A virus genes [16], [17]. By the end of 1999 these TR H3N2 viruses were widespread in the US swine population and were first reported in Canadian pigs and turkeys in 2005 [6].

Approximately 45% of the turkey production in Canada is located in Ontario, often in areas that are densely populated with both swine and turkey farms. Since 2005, drops in egg production in breeder turkeys attributed to and coinciding with the circulation of TR H3N2 viruses in adjacent pig farms [6] has plagued turkey producers in Southern Ontario. Others have reported similar reproductive problems in female turkeys in association with TR H3N2 infection [18], [19], [20], [21]. As was previously suggested for the situation in the US [18], the close proximity of swine and turkey farms appears to play a significant role in the epidemiology of TR H3N2 influenza virus infection of turkeys.

In a previous study [13], we genetically characterized pandemic H1N1 2009 viruses isolated from breeder turkeys that had an associated drop in egg production. In this paper, we antigenically and genetically characterized three unique TR H3N2 influenza viruses that were isolated from three breeder turkey flocks at different geographic locations in Southern Ontario at different times during 2011.

## Materials and Methods

### Clinical submissions

This study involved a field outbreak of influenza A virus in turkeys. The owners of the animals associated with this study have read and approved this manuscript but wish to remain anonymous. Virus isolation procedures involving embryonating chicken eggs were in compliance with Canadian Council for Animal Care guidelines. The chicken embryos were inoculated at 9 days of gestation and fluids harvested 5 days afterwards.

All three diagnostic submissions were comprised of tracheal and cloacal swabs. They originated from three geographically separate breeder turkey farms owned by the same company in Southern Ontario that had experienced significant drops in egg production. Two of three flocks were vaccinated with an inactivated A/Mallard Duck/MN/79/79 (H3N4) oil emulsion vaccine and had detectable antibody titers before exposure to field virus. The original samples concerning this study were collected by the company veterinarian and submitted to the provincial veterinary diagnostic laboratory (Animal Health Laboratory, University of Guelph). Diagnostic test results along with the samples were forwarded to the National Centre for Foreign Animal Disease (NCFAD) as part of a federally mandated disease surveillance program. Additional work was carried out at the company's request to determine the reason for vaccine failure. The owners of the birds provided NCFAD with the convalescent sera collected from sick turkeys and the sera collected from H3N4 vaccinated turkeys prior to field virus as part of a follow up study aimed at determining the reason for vaccine failure.

Reference antisera to A/Perth/16/2009 (H3N2) produced in ferrets was carried out under Animal Use Document H-07-13 “Preparation of reference antisera to various strains of live human influenza virus in ferrets” approved by The Canadian Science Centre for Human & Animal Health Animal Care Committee in compliance with Canadian Council for Animal Care guidelines. The remaining antisera were prepared under Animal Use Document C-08-002 “Production of antisera to avian influenza viruses and avian paramyxoviruses” approved by the same Animal Care Committee.

The chicken red blood cells are obtained on a weekly basis from the Canadian Food Inspection Agency's Ontario Laboratory Fallowfield specific-pathogen-free flock under animal use document ACC 11-03 “Blood collection from farm animals”, that was approved by the institutional Animal Care Committee in compliance with Canadian Council for Animal Care guidelines. The turkey red blood cells were purchased from LAMPIRE Biological Laboratories, Inc. P.O. Box 270 Pipersville, PA, USA.

#### 1^st^ submission (FAV-003)

On February 24, 2011 tracheal and cloacal swabs from a breeder turkey flock of 15,000 that was exhibiting a sudden drop in egg production with no other apparent clinical signs were submitted to the Animal Health Laboratory (AHL), University of Guelph. Turkey hens from this flock had not been vaccinated with the inactivated H3N4 vaccine mentioned above. A H3N2 subtype influenza A virus was identified by molecular means and on February 25 tracheal and cloacal swabs were forwarded to the National Centre for Foreign Animal Disease (NCFAD), Winnipeg for virus isolation and further characterization. Sixteen convalescent serum samples were submitted at a later time point as part of the follow up investigation.

#### 2^nd^ submission (FAV-009)

On June 6, 2011, 34-week-old turkey hens from a different geographical location exhibited a 10% drop in egg production with no other apparent clinical signs. A H3 subtype influenza A virus was identified by molecular means at AHL, University of Guelph and tracheal and cloacal swabs were forwarded to NCFAD, Winnipeg for confirmation and further characterization. These turkeys had been previously immunized with an inactivated H3N4 vaccine. Follow up serum samples were not obtained from birds in this flock.

#### 3^rd^ submission (FAV-010)

On June 17, 2011, a flock from a third geographical location exhibited a 20% drop in egg production with no other clinical signs. These turkeys had received the inactivated H3N4 vaccine. Tracheal and cloacal swabs were forwarded to NCFAD on June 20^th^ for confirmation. Nineteen serum samples collected before the turkeys exhibited the drop in egg production were submitted as part of a follow up investigation.

### RNA extraction from swab samples

Tracheal and cloacal swab specimens were clarified by centrifugation, 50 µl of sample was spiked with an exogenous internal control (for evaluating nucleic acid extraction efficiency and presence of PCR inhibitors during RT-PCR) and RNA was extracted with the MagMAX™-96 Total RNA Isolation Kit using the MagMax 96-well robotic system (Applied Biosystems/Ambion, Austin, Texas).

### Real time RT-PCR

Total RNA extracted from the swab specimens were tested using the M1 gene specific real-time reverse transcription polymerase chain reaction (RRT-PCR) assay [22] and the modified version of this assay developed at NCFAD and described previously [9].

### Virus isolation

Virus isolation was carried out by inoculating the allantoic cavity of 9-day-old specific-pathogen-free (SPF) embryonating chicken eggs with clarified and antibiotic treated swab samples. Embryos were monitored daily for mortality. Amnio-allantoic fluid (AAF) from dead embryos as well as from embryos at the end of 1^st^ and 2^nd^ passages were harvested and tested for the presence of hemagglutinating agents with chicken red blood cells (CRBC). The AAF were also tested for the presence of influenza A nucleic acids by a real-time RT-PCR assay as described above to exclude presence of influenza A viruses that did not hemagglutinate CRBC. All submissions underwent up to two passages before being considered negative.

### Hemagglutination and hemagglutination inhibition tests

Hemagglutination and hemagglutination inhibition (HI) tests were carried out using standard procedures. For the hemagglutination test, AAF was tested for the presence of hemagglutinating agents using CRBC or turkey red blood cells (TRBC). Antigenic characterization of the new isolates was performed by HI assay using a panel of reference antisera prepared against the 16 known HA subtypes of influenza A viruses. Two fold serial dilutions of each reference antiserum were mixed with 4 HA units of each virus, followed by the addition of 0.5% (v/v) suspensions of CRBC or TRBC. The reciprocal of the highest dilution of serum that completely inhibited hemagglutination was considered the HI titre. The reference antiserum that produced the highest HI titer indicated the HA subtype of the isolate.

### Immune plaque reduction virus neutralization assay

Immune plaque reduction virus neutralization (IPRVN) assay was carried out using MDCK cells grown overnight to confluency in 96-well tissue culture plates (Corning, USA). Virus neutralization was carried out using a constant amount of H3N2 virus (100 plaque forming units) mixed with equal volumes of 2-fold serial dilutions (starting 1∶20) of convalescent sera collected from diseased turkeys, field sera collected from turkeys immunized with the H3N4 vaccine prior to exposure, as well as a panel of reference H3 antisera. After 1 hr of incubation at 37°C, the virus/antisera mixtures were applied to the MDCK cell monolayers and incubated for an additional 1 hr at 37°C. The virus/antiserum mixture was then replaced with DMEM containing 0.2% (w/v) bovine serum albumin and 1.5% carboxymethyl cellulose (Sigma). The cells were incubated at 37°C in a humidified atmosphere of 5% CO_2_ for 48 hrs after which they were fixed in 10% formalin solution in PBS. Cells were then permeablized with 20% acetone in PBS, washed with PBS-Tween and then primed with anti-influenza nucleoprotein monoclonal antibody [9] for 1 hr. After 3 washes with PBS-Tween solution, the cells were allowed to incubate with HRP-conjugated goat anti-mouse secondary antibody (Jackson Immunoresearch) for 1 hr. Finally, plaques were stained with TrueBlue substrate (KPL, Gaithersburg, MD) and visualized under the microscope and counted.

The following viruses were used in the IPRVN assay: A/Turkey/ON/FAV-003/2011 (H3N2), A/Turkey/ON/FAV-009/2011 (H3N2), A/Mallard/QC/2323-66/2006 (H3N2), A/Duck/BC/7846/2006 (H3N8) and A/Turkey/BC/1529-3/2005 (H3N2) a TR virus isolated from domestic turkeys. Reference antisera raised against A/Turkey/BC/1529-3/2005 (H3N2), A/Duck/BC/7846/2006 (H3N8) and A/Perth/16/2009 (H3N2) (donated by Dr Yan Li, National Microbiology Laboratory, Public Health Agency of Canada) along with field serum samples collected from turkeys that were vaccinated with A/Mallard Duck/MN/79/79 (H3N4) prior to exposure to wild type H3N2 virus and convalescent serum samples collected from turkeys exposed to A/Turkey/ON/FAV-003/2011 (H3N2), but that were not vaccinated with the H3N4 vaccine were assessed by IPRVN assay.

### Amplification, cloning and sequencing of full influenza A gene segments

Viral RNA was extracted from infectious AAF collected from embryonating chicken eggs. Total RNA was extracted as described above using the MagMAX™-96 Total RNA Isolation Kit. Full-length influenza A gene segments were RT-PCR amplified using universal influenza A primers [23], ligated into the pCR4®-TOPO® cloning vector (Invitrogen) which was then used to transform One-Shot TOPO10 *E. coli* (Invitrogen). Bacterial clones were screened by PCR and plasmids from clones that contained the genes of interest were used for sequencing as described previously [24].

### Phylogenetic analysis

Phylogenetic analysis was performed as described previously [24]. Briefly, the full HA and NA nucleotide sequences and the reference virus sequences retrieved from GenBank were aligned initially with the Megalign program (DNASTAR, Madison, WI), using the Clustal V alignment algorithm. Generation of phylogenetic trees was performed by using molecular evolutionary genetics analysis version 4 (MEGA 4). Phylogenetic trees were generated with the close-neighbor joining and 500 bootstrap replicate options of the maximum parsimony method.

### Crystal structure manipulations

The amino acid sequences of the HA1 subunit of A/Turkey/ON/FAV-003/2011 (H3N2) and A/Turkey/ON/FAV-009/2011 (H3N2) were aligned with those of A/Mallard Duck/MN/1979 (H3N4) and A/Swine/ON/33853/2005 (H3N2) to identify amino acid substitutions within the five antigenic sites (A, B, C, D and E). The HA crystal structure of the H3 subtype influenza virus, A/duck/Ukraine/1963 (PDB 1MQL) [25] was used as reference. Molecular graphics images were produced using PyMOL (http://www.pymol.org). Resulting images were imported into Adobe Photoshop and assembled with Adobe Illustrator (Adobe).

## Results

### Clinical submission # 1 (FAV-003)

All 5 swab specimens that were submitted to NCFAD on February 25, 2011 tested positive with the influenza A matrix real time RT-PCR assay developed by USDA [22]. Samples were inoculated into embryonating SPF chicken eggs with only one yielding virus after 2^nd^ passage which did not hemagglutinate CRBC. The presence of virus in AAF was confirmed by influenza A matrix real time RT-PCR assay. Real time RT-PCR and virus isolation results for this and the other 2 submissions are summarized in Table 1.

**Table 1:** Influenza A matrix gene real time RT-PCR and virus isolation results for the swab specimens received from the three turkey farms.

Submission Number	Number of swabs	Matrix real time RT-PCR		Isolation in embryonating chicken eggs
		Spackman	Modified	
FAV-003	5	5/5	ND	1/5
FAV-009	6	1/6	6/6	1/6
FAV-0010	7	ND	5/6	3/6

ND = not determined.

Turkeys in this barn had not been immunized with the inactivated H3N4 vaccine. Convalescent serum samples (n = 16) submitted from this farm tested positive on HI assay using a panel of reference H3 viruses. The results, which are summarized in Table 2, show that these sera reacted with the Cluster IV TR H3N2 virus A/Turkey/BC/1529/2005 as well as A/Mallard/QC/2223-66/2006 which was isolated from a mallard duck in Quebec in 2006.

**Table 2:** Hemagglutination inhibition titres of pre-exposure serum samples submitted from turkeys vaccinated with H3N4 (FAV- 0010) and convalescent sera collected from turkeys exposed to triple reassortant H3N2 field virus (FAV-003).

Serum	A/Tk/BC/01529/2005(H3N2)	A/Mallard/QC/2323-6/2006(H3N2)	A/Tk/ON/FAV003/2011(H3N2)	A/Tk/ON/FAV009/2011(H3N2)	A/Tk/ON/FAV0010/2011(H3N2)
**FAV-010 Serum Submission^1^**					
1	Neg	Neg	Neg	Neg	Neg
2	16	256	8	16	32
3	Neg	32	Neg	Neg	8
4	16	256	8	8	16
5	Neg	32	Neg	Neg	32
6	32	64	8	16	32
7	Neg	256	8	8	32
8	Neg	256	Neg	8	32
9	Neg	32	Neg	Neg	4
10	Neg	64	Neg	Neg	Neg
11	8	256	8	8	32
12	Neg	256	8	8	32
13	Neg	128	Neg	Neg	32
14	Neg	512	Neg	8	32
15	Neg	1024	8	16	64
16	8	16	Neg	4	32
17	Neg	32	Neg	Neg	8
18	Neg	32	Neg	Neg	16
19	Neg	64	Neg	Neg	16
**FAV-003 Serum submission**					
20	>8192	64	>8192	256	1024
21	>8192	512	>8192	512	1024
22	>8192	512	>8192	512	4096
23	4096	128	>8192	256	2048
24	2048	64	4096	512	4096
25	>8192	4096	4096	512	2048
26	>8192	4096	4096	1024	4096
27	4096	64	>8192	256	1024
28	>8192	2048	4096	1024	2048
29	>8192	512	1024	512	4096
30	4096	128	>8192	512	2048
31	>8196	256	>8196	512	1024
32	4096	128	4096	512	2048
33	>8192	64	>8192	512	2048
34	>8192	2048	>8192	1024	>8192
35	>8192	128	>8192	512	>8192

1 Sera from submission FAV-010 tested negative for antibodies to pH1N1 by hemagglutination-inhibition assay.

### Clinical submission # 2 (FAV-009)

Three tracheal and 3 cloacal swab specimens from this farm tested negative using the influenza A matrix real time RT-PCR assay originally described by USDA [22]. When these were re-tested using a modified version of this assay designed to have an increased analytic sensitivity for the matrix gene segment found in pH1N1 2009 [9], 5 out of 6 of the samples tested positive. However, a virus which hemagglutinated CRBC and TRBC was isolated from only one of the tracheal swab specimens.

### Clinical submission # 3 (FAV-010)

The swab specimens from this submission also tested negative when the real time RT-PCR assay targeting the matrix gene as originally described previously [22] was used; however, 5 out of 6 swabs tested positive using the modified version of this assay and 3 out of 6 samples yielded isolates that hemagglutinated CRBC and TRBC. These turkeys had been immunized with an inactivated H3N4 vaccine prior to exhibiting a drop in egg production. Nineteen serum samples that had been collected following vaccination but prior to the drop in egg production were tested using a panel of reference H3 viruses. The results, which are summarized in Table 2, show that the sera from this farm (submission FAV-010) reacted most strongly with the H3N2 virus isolated from a mallard duck in 2006. In contrast, low to negative titers were observed against the viruses that were isolated from these 3 submissions after the birds exhibited a drop in egg production as well as against a Cluster IV TR H3N2 virus that was isolated from turkeys in 2005.

### Virus identification and typing

For the single isolate from submission FAV-003, virus HA subtyping was done using molecular techniques as this virus did not hemagglutinate CRBC. For this purpose, the full HA gene segment was amplified, cloned and sequenced. NA subtyping was also determined by molecular means using the universal primers described by Hoffman et al. [23]. Sequencing of the full HA and NA gene segments confirmed this virus to be H3N2. Since isolates from submissions FAV-009 and FAV-0010 yielded viruses that hemagglutinated CRBC and TRBC, the HA subtyping was done by HI assay using a panel of reference antisera developed against all known 16 HA subtypes. Isolates from both submissions reacted with rabbit polyclonal serum developed against A/Turkey/BC/01529/2005 (H3N2). Neuraminidase typing was done by RT-PCR, followed by sequencing as described above and isolates from both submissions were typed as N2.

### Genetic characterization of H3N2 isolates

The 8 gene segments for the three H3N2 isolates were amplified, cloned and sequenced. The sequence data for the H3N2 viruses isolated in this study were deposited in GenBank (FAV-003 acc # JN683626-33; FAV-009 acc # JN683634-41 and FAV-0010 acc # JN706697-704). Genetic relatedness of each gene segment from each of the isolates from the three farms was compared with other published influenza A sequences using the basic alignment search tool (BLAST) from the GenBank database. Based on the BLAST search, we were able to identify two genetically distinct H3N2 viruses. The single isolate from submission FAV-003 was identified as a triple reassortant virus containing gene segments of avian (PB2, PA), human (PB1, HA, NA) and swine (NP, M, NS) influenza virus origin. The two virus isolates from submissions FAV-009 and FAV-0010 contained a constellation of genes that has not been previously described. Gene segments PB2, PA, NP and M were from pandemic H1N1 2009 while the remaining gene segments (PB1, HA, NA and NS) originated from the TR H3N2 viruses that were isolated from pigs in the USA beginning in 1998 and in Canada beginning in 2005. The percentage of genetic relatedness of the three H3N2 isolates to other published influenza viruses in the NCBI database are summarized in Table 3.

**Table 3:** Top BLAST matches of the 8 gene segments from FAV-003 and FAV-009 obtained from NCBI influenza A virus nucleotide database.

Segment		A/Tk/ON/FAV003/2011(H3N2)	A/Tk/ON/FAV009/2011(H3N2)
PB2	NA	99% A/swine/QC/1840-2/2009(H3N2)	99% A/Ontario/315637/2009(H1N1)
	AA	99% A/swine/QC/1840-2/2009(H3N2)	99% A/Australia/24/2009(H1N1)
PB1	NA	99% A/swine/QC/1698-1/2009(H3N2)	99% A/swine/Minnesota/66853/2006(H3N2)
	AA	99% A/swine/QC/1840-2/2009(H3N2)	99% A/turkey/BC/1529-3/2005(H3N2)
PA	NA	99% A/swine/QC/1698-1/2009(H3N2)	99% A/Ontaio/9739/2009(H1N1)
	AA	99% A/swine/QC/1840-2/2009(H3N2)	99% A/Canada-MB/RV2023/2009(H1N1)
HA	NA	99% A/swine/QC/1698-2/2009(H3N2)	99% A/swine/QC/1268883/2010(H3N2)
	AA	98% A/swine/QC/1840-2/2009(H3N2)	99% A/swine/QC/1268883/2010(H3N2)
NP	NA	99% A/swine/QC/1698-1/2009(H3N2)	99% A/swine/Taiwan/CH-1204/2004(H1N1)
	AA	99% A/swine/QC/1697-1/2009(H3N2)	98% A/Regensburg/D6/2009(H1N1)
NA	NA	99% A/swine/QC/1698-2/2009(H3N2)	98% A/Tk/BC/1529-3/2005(H3N2)
	AA	99% A/swine/QC/1698-5/2009(H3N2)	98% A/Ontario/RV1273/2005(H3N2)
M	NA	99% A/swine/QC/1698-5/2009(H3N2)	99% A/Taiwan/126/2009(H1N1)
	AA	99% A/swine/QC/1840-2/2009(H3N2)	100% A/Ontario/RV1527/2009(H1N1)
NS	NA	98% A/swine/QC/1698-1/2009(H3N2)	98% A/Tk/OH/313053/2004(H3N2)
	AA	97% A/swine/QC/1698-1/2009(H3N2)	97% A/Sw/N.Carolina/02023/2008(H1N1)

### Phylogenetic characterization of the H3N2 isolates

**Figure 1:**
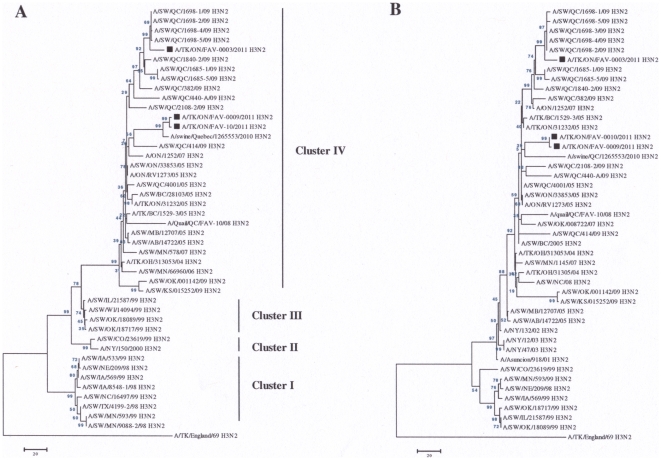
Phylogenetic analysis of the HA and NA genes. The HA (a) and NA (b) gene segments of the unique TR H3N2 viruses isolated from turkeys were compared with other TR H3N2 viruses from turkey (open circle), quail (open diamond) and pig (open triangles) that were previously sequenced by our laboratory [24].

Phylogenetic analysis of the HA and NA genes of the virus isolated from submission FAV-003 showed that they clustered with other TR H3N2 viruses that were isolated from pigs in Quebec in 2009 (Fig. 1). The HA (Fig. 1A) and NA (Fig. 1B) genes from the two virus isolates from submissions FAV-009 and FAV-0010 showed a close evolutionary relationship with a TR H3N2 virus that was isolated from pigs in the province of Quebec in 2010 (Fig. 1).

### Antigenic characterization of the newly isolated viruses

We compared the ability of the three newly isolated TR H3N2 viruses to react with different reference H3 antisera as well as field sera that had been collected from turkeys that were vaccinated with the inactivated H3N4 vaccine (submission FAV-0010) and convalescent serum collected from turkeys following H3N2 exposure (submission FAV-003). Polyclonal antisera raised against the TR H3N2 virus A/Turkey/BC/1529-3/2005 and the human seasonal H3N2 virus A/Perth/16/2009 were able to neutralize all three of the newly isolated viruses. In contrast, polyclonal antiserum raised against A/Duck/BC/7846/2006 (H3N8) poorly neutralized the TR H3N2 viruses that were isolated from the turkeys in this study. The sera collected from H3N4 vaccinated turkeys (submission FAV-0010) showed strong cross-neutralizing activity against A/Mallard/QC/2323-66/2006 (H3N2), but weak activity (titer = 1/40) against the three newly isolated TR H3N2 viruses as well as an earlier TR H3N2 virus from turkeys (A/ Turkey/BC/1529-3/2005). The convalescent sera collected from turkeys from submission FAV-003 cross-neutralized all three of the newly isolated TR H3N2 viruses as well as A/Mallard/QC/2323-66/2006 (H3N2) and A/Turkey/BC/1529-3/2005 (H3N2). This indicated that the flock might have been previously exposed to H3 viruses of avian and swine TR H3N2 origin. The virus neutralization titer was higher (>2560) against an isolate from the same farm (FAV-003). Results for HI assays (Table 4) show similar cross-reactivity patterns as were observed with the virus neutralization assay (Table 5).

**Table 4:** Antigenic characterization of triple reassortant H3N2 viruses isolated from turkeys by hemagglutination-inhibition assay using turkey red blood cells and various reference H3 antisera.

Polyclonal Antisera	Viruses						
	Dk/BC/7846/06	Tk/BC/1529/05	Dk/ON/05/00	Perth/16/09	Tk/ON/FAV003/11	Tk/ON/FAV009/11	Tk/ON/FAV010/11
Rabbit 1anti- A/Dk/BC/7846/06(H3N8)	2048	32	ND	ND	32	16	16
Rabbit 2anti- A/Tk/BC/1529/05(TR H3N2)	ND	4096	32	ND	2048	128	256
Goat 3anti- A/Dk/ON/05/00(TR H3N2)	ND	32	2048	ND	32	16	16
Ferret 4anti- A/Perth/16/09(H3N2)	ND	128	ND	640	512	256	128
Negative Rabbit Serum	0	0	0	0	0	0	0

**Table 5:** Summary of cross neutralization assay results as determined by IPRVN of TR H3N2 viruses isolated from turkeys using various reference H3 antisera.

PolyclonalAntisera	Viruses				
	A/Tk/BC/15293/05 (TR H3N2)	A/Dk/BC/7846/06 (H3N8)	A/Tk/ON/FAV003/11 (H3N2)	Tk/ON/FAV009/11(H3N2)	A/Mal/QC/2323-66/06 (H3N2)
A/Tk/BC/1529-3/05(TR H3N2)	2560	ND	640	640	40
A/Dk/BC/7846/06(H3N8)	80	2560	40	40	2560
A/Perth/16/09(H3N2)	1280	ND	640	1280	40
FAV-010 (#1)H3N4 vaccinated	40	ND	40	40	1280
FAV-010 (#2)H3N4 vaccinated	40	ND	40	40	2560
Convalescent serum FAV-003 (#1)	2560	ND	2560	2560	1280
Convalescent serum FAV-003 (#2)	1280	ND	1280	1280	2560

ND – Not Done.

### Molecular characterization of the hemagglutinin protein

To determine the level of genetic relatedness between the three H3N2 turkey isolates, the full hemagglutinin protein (HA0) and the 328 residues of the HA1 subunit were subjected to pairwise amino acid identity comparisons with the A/Mallard duck/79/79 (H3N4) vaccine strain [18] and a prototype cluster IV TR H3N2 virus A/Swine/ON/33853/2005 [8] using the ALIGN tool (NCBI sever). Results of this comparison are summarized in Table 6.

**Table 6:** Percent similarities in the amino acid residues of the HA0 and HA1 of the three H3N2 isolates from turkeys compared to a prototype cluster IV TR H3N2 strain.

	A/Tk/ON/FAV003/2011		A/Tk/ON/FAV010/2011		A/Sw/ON/33853/2005	
Isolate	HA_0_	HA_1_	HA_0_	HA_1_	HA_0_	HA_1_
A/Tk/ON/FAV009/2011	94%	91%	-	-	-	-
A/Sw/ON/33853/2005	97%	95%	96%	95%	-	-
A/Tk/ON/FAV010/2011	94%	91%	99%	99%	96%	95%
A/M.duck/MN/79/1979	ND	79%	ND	80%	ND	80%

ND = Not done [full HA sequence of A/Mallard duck/MN/79/1979 (H3N4) was not available].

The predicted amino acid identities of the HAl subunit (excluding the signal peptide) between the H3N4 vaccine strain and FAV-003 was 79% and between the H3N4 vaccine strain and FAV-009 or FAV-0010 was 80%. By comparison, the predicted amino acid identity between a prototype H3N2 (A/Sw/ON/33853/2005) virus from cluster IV and the three turkey isolates was 95%. When the turkey isolates were compared with each other, the predicted amino acid identity decreased to 91%.

**Figure 2:**
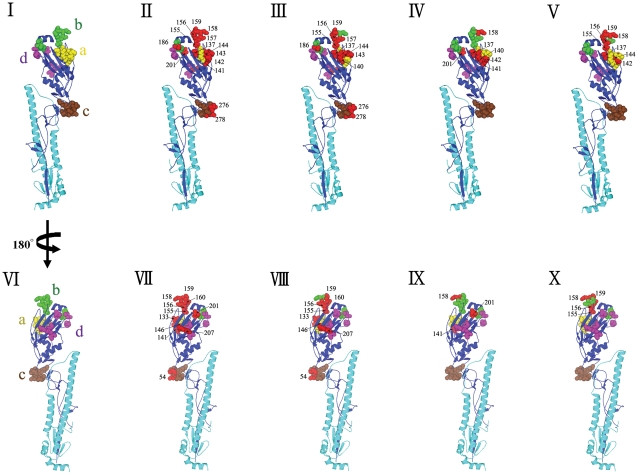
Locations of the amino acid alterations at the major antigenic sites of HA1 molecule in the H3 crystal structure. (I) Side view of a HA monomer in cartoon format with major antigenic sites A, B, C and D shown in spheres (II, III, IV, and V??amino acid changes identified at the major antigenic sites. The locations of changed amino acids are indicated and colored in red. (VI, VII, VIII, IX, and X) Back view of panels I, II, III, IV, and V, respectively. The view in panels I to V is rotated 180° along Y-axis.TK/ON/FAV-003/2011 vs. DK/MN/1979 (II and VII); TK/ON/FAV-009/2011 vs. DK/MN/1979 (III and VIII); TK/ON/FAV-003/2011 vs. SW/ON/33853/2005 (IV and IX); TK/ON/FAV-009/2011 vs. SW/ON/33853/2005 (V and X).

**Figure 3:**
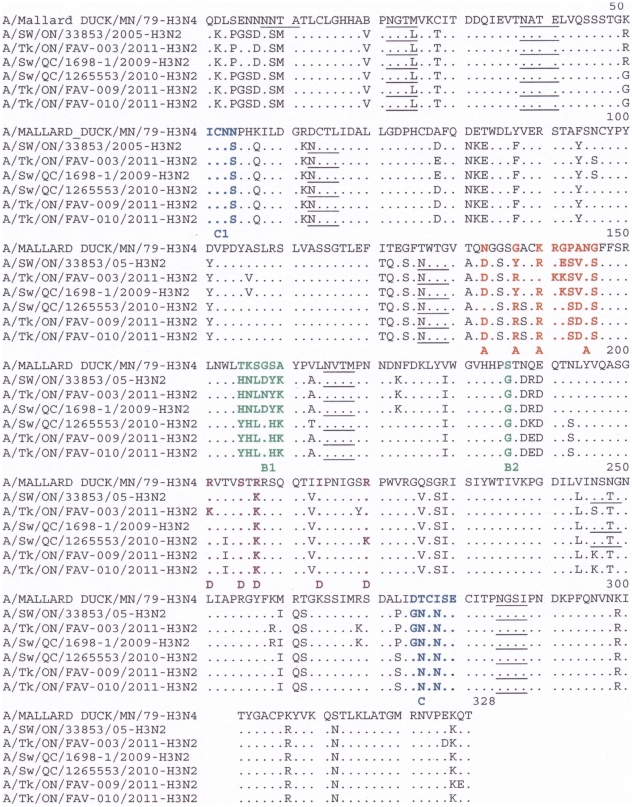
Alignment of the H3 HA1 amino acid sequences (without signal peptide). Amino acids of the HA1 subunit of the three unique turkey isolates, the duck H3N4 vaccine strain, a prototype cluster IV TR H3N2 virus (A/SW/ON/33853/2005) and phylogenetically related isolates A/Sw/QC/1265553/2010 (H3N2) and A/SW/QC/1698-1/2009 (H3N2) were aligned. Residues shown in red, green, blue and purple represent previously identified antigenic sites A, B, C and D respectively. Potential glycosylation sites are underlined.

To determine whether the amino acid changes occurred in any of the previously identified [18], [26] 5 antigenic sites (A, B, C, D and E), we aligned the amino acid sequences of the 3 turkey isolates and compared them with the H3N4 vaccine strain and a prototype cluster IV H3N2 virus and marked the antigenic sites as presented in Fig. 2 and Fig. 3. Compared to the vaccine strain, FAV-003 and FAV-009/10 had 19 and 16 amino acid changes within the major antigenic sites respectively. The triple reassortant isolate from the FAV-003 submission contained at least 8 amino acid differences in HA1 when compared to the swine H3N2 virus that it was most closely related phylogenetically. In contrast, FAV -009/10 isolates had 5 amino acid differences in the 328 amino acid residues of the HA1 subunit when compared with the phylogenetically related A/Sw/QC/1265553/2010 (H3N2) virus. The turkey isolates from this study possessed some unique changes at antigenic site B; in the prototype cluster IV virus, amino acids at position 155 – 160 (HNLDYK) were changed to HNLNYK in the virus isolated from submission FAV-003 and YHLGHK in the viruses isolated from submissions FAV-009/10. The changes in antigenic site A for FAV-009/10 were almost identical to those of A/Sw/QC/126553/2010 (H3N2), but 132N was substituted with 132D. The receptor binding site (RBS) of the influenza virus HA is a conserved pocket of amino acids surrounded by antigenically variable antibody binding sites [27]. The RBS of the viruses examined in this study were mostly conserved; the turkey viruses had the amino acids Y98, G134, S136, W153, H183, Y195,G225 and S227 which was the same for the prototype TR H3N2 cluster IV virus and the H3N4 vaccine strain. Notable changes were E190D, Q226V and G228S which were found in all 3 turkey viruses when compared with the H3N4 vaccine strain.

To predict N-linked glycosylation sites (Asn-X-Ser/Thr, where X is any amino acid except Pro), we used the NetNGlyc 1.0 Server. Based on this, the H3N4 vaccine strain had 5 glycosylation sites at positions 8, 22, 38, 165 and 285 as described previously [18]. However, the three turkey isolates from this study had 6 potential N-linked glycosylation sites at positions 22, 38, 63, 126, 165 and 285 (underlined in Fig. 2). These sites were also the same for other phylogenetically related triple reassortant viruses, but the three turkey isolates lacked one additional potential glycosylation site at residue 246. This site was also present in the prototype cluster IV virus and other recent and closely related swine isolates from Quebec.

## Discussion

In a previous report, we isolated pandemic H1N1 2009 virus from a breeder turkey flock that exhibited respiratory illness and a drop in egg production [13]. In this report, we describe the genetic and antigenic characterization of unique TR H3N2 viruses bearing gene segments from 2009 pandemic H1N1 (PB2, PA, NP and M) and TR H3N2 (PB1, HA, NA and NS) viruses, the latter of which have been circulating in swine and soon after turkey populations in the USA since 1998 [16] and in Canada since 2005 [8]. To our knowledge, this is the first report of the isolation of pH1N1/TR H3N2 reassortant viruses from a domestic poultry species.

Phylogenetic analysis of the HA and NA genes from FAV-009 and FAV-0010 showed that they are closely related to A/Swine/QC/126553/2010 (H3N2) and A/Swine/QC/1268883/2010 (H3N2) which were isolated from pigs with respiratory illness in Quebec. The latter H3N2 isolate from swine also has pandemic H1N1 internal genes [28], but of a different assemblage than those found in FAV-009 and FAV-010. The other TR H3N2 virus (FAV-003) was closely related to other TR H3N2 viruses that were isolated from pigs in 2009 in Quebec. Although phylogenetic analysis shows that these viruses were likely introduced to turkeys from pigs, we were not able to find any epidemiologic links between the farms.

In contrast to the chicken reproductive tract that contains only α2-3-linked sialic acids receptors, turkeys may contain both α2-3-linked and α2-6-linked sialic acids receptors in their reproductive tract which could be involved in the attachment and replication of human and swine-like influenza viruses [19], [20]. Amino acids at position 226 and 228 in the HA1 subunit of H3 subtype influenza viruses were shown to be necessary for the switch in receptor specificity from α2-3-linked to α2-6-linked sialic acids and adaptation from avian to mammalian hosts [29]. All three turkey isolates described here had mutations in these 2 amino acid positions (Q226V and G228S) that are associated with the change from α2-3 to α2-6 specificity. The drop in egg production in these turkeys is most likely associated with virus replication in their reproductive tracts.

Despite the fact that pigs are normally viewed as “mixing vessels” for the generation of reassortant influenza A viruses, we cannot exclude the possibility that the reassortment between TR H3N2 and pH1N1 took place in turkeys, even though sera from submission FAV-0010 gave negative HI results when using pandemic H1N1 antigen (data not shown). Nevertheless, the results described here should be of concern, considering the reassortment capacity of this virus and the susceptibility of turkeys to influenza viruses of H1 to H16 subtypes. According to Nobusawa et al. [30], a point mutation that resulted in a Glu to Asp substitution at amino acid position 190 (E190D) in the HA protein of A/Aichi/51/92 was responsible for the loss of the ability to bind to CRBC. All three isolates described in this study were tested for their ability to agglutinate CRBC and TRBC. A/Tk/ON/FAV-003/2011 did not agglutinate CRBC, but could agglutinate TRBC. The other two isolates had better hemagglutination results with TRBC than CRBC. All 3 isolates had amino acid change at E190D, but the only isolate that didn't agglutinate CRBC was from FAV-003. Therefore, multiple amino acid changes involving the HA protein are likely necessary to change the receptor binding specificity. Nakajima et al. 2003 [31] suggested that the effect of hemadsorption activity of an amino acid change on the HA protein primarily depends on the position rather than the species of substituted amino acid. They showed that mutation of the amino acid at position 156 from lysine to glutamic acid, asparagine, glutamine, or isoleucine was shown to be associated with the loss of hemadsorption activity. FAV-003 has asparagine at this position and this might have had the negative effect on hemagglutination activity, but the other 2 isolates with hemagglutination activity had histidine at this position.

The hemagglutinin protein of H3 influenza viruses has accumulated a number of glycosylation sites during its evolution over the past 40 years [32]. Glycosylation appears to be one way by which a virus can mask its epitopes and evade detection by the host's immune system. In addition, some studies have associated increasing glycosylation with reduced virulence [32], [33], [34]. Although the three turkey isolates from this study shared 6 potential N-linked glycosylation sites at position 22, 38, 63, 126, 165 and 285 with the closely related cluster IV viruses of swine, they lacked a potential glycosylation site at position 246.

In Canada, the only approved vaccine for turkeys against H3N2 viruses is the inactivated H3N4 vaccine made from a strain isolated from a mallard duck in 1979. Although, two of the breeder turkey flocks in this study were immunized with this vaccine, the field strains described here were able to infect the turkeys and cause a loss in egg production. The fact that the sera collected from H3N4 vaccinated turkeys were not able to neutralize any of the newly isolated H3N2 viruses described in this study confirms the poor efficacy of this vaccine. We believe the vaccine failure was associated with amino acid substitutions in the globular head domain of the HA1 subunit which contains the immunodominant antigenic sites. The percent amino acid identity of the HA1 between the H3N4 vaccine strain and FAV-003 was 79% and with FAV-009 or FAV-0010 – 80%.

Previous studies have also shown that mutations in the 5 immunodominant antigenic sites located on the globular head of the HA1 subunit play a key role in virus escape from host immune pressure as a result of accumulated conformational changes [18], [30], [35]. The amino acid substitutions coupled with changes associated with the appearance of oligosaccharide side chains in the globular head region are responsible for the generation of antigenic variants [36]. The HA1 of the viruses from FAV-003 and FAV-009/10 had 19 and 16 amino acid changes respectively at these major antigenic sites when compared with the H3N4 vaccine strain. In addition, the isolate from the FAV-003 submission contained at least 8 amino acid differences in HA1 when compared to the swine H3N2 virus that it was most closely related to phylogenetically. According to Wilson and Cox [35], drifting antigenic variants of epidemiologic importance could emerge if changes in the five antigenic sites involve more than 4 amino acids and the changes are located in more than 2 of the 5 antigenic sites. The major changes in these newly isolated viruses were associated with antigenic sites A and B (Fig. 2 and Fig. 3), which are near the receptor binding site and often play a key role in role in escape from neutralizing antibodies.

In both humans and domestic animals influenza variants frequently emerge as a result of point mutations in the HA gene, resulting in new variants that escape the host immune response. The vaccine failure described in this study is not surprising; selection of viruses for animal influenza vaccines should be based on the results of recent epizootologic, virologic and immunologic surveillance results. Two of the H3N2 isolates characterized in this study contained a unique combination of genes derived from pandemic H1N1 (2009) which underscores the need for continuous surveillance and monitoring of the genetic changes of influenza A viruses circulating in domestic animals to not only aid in the selection of the most appropriate vaccine strains but to track the evolution of viruses that might pose new threats to human and animal health.

## References

[1] Tang Y, Lee CW, Zhang Y, Senne DA, Dearth R (2005). Isolation and characterization of H3N2 influenza A virus from turkeys.. Avian Dis.

[2] Webster RG, Bean WJ, Gorman OT, Chambers TM, Kawaoka Y (1992). Evolution and ecology of influenza A viruses.. Microbiol Rev.

[3] Matrosovich M, Tuzikov A, Bovin N, Gambaryan A, Klimov A (2000). Early alterations of the receptor-binding properties of H1, H2, and H3 avian influenza virus hemagglutinins after their introduction into mammals.. J Virol.

[4] Yassine HM, Khatri M, Lee CW, Saif YM (2010). Characterization of an H3N2 triple reassortant influenza virus with a mutation at the receptor binding domain (D190A) that occurred upon virus transmission from turkeys to pigs.. Virol J 30;.

[5] Holmes EC, Ghedin E, Miller N, Taylor J, Bao Y (2005). Whole-Genome Analysis of Human Influenza A Virus Reveals Multiple Persistent Lineages and Reassortment among Recent H3N2 Viruses.. PLoS Biol.

[6] Shil P, Chavan S, Cherian S (2011). Molecular basis of antigenic drift in Influenza A/H3N2 strains (1968–2007) in the light of antigen antibody interactions.. Bioinformation.

[7] Memoli MJ, Jagger BW, Dugan VG, Qi L, Jackson JP (2009). Recent human influenza A/H3N2 virus evolution driven by novel selection factors in addition to antigenic drift.. J Infect Dis.

[8] Olsen CW, Karasin AI, Carmen S, Li Y, Bastien N (2005). Triple reassortant H3N2 influenza A viruses, Canada.. Emerg Infect Dis.

[9] Weingartl HM, Berhane Y, Hisanaga T, Neufeld J, Kehler H (2010). Genetic and pathobiologic characterization of pandemic H1N1 2009 influenza viruses from a naturally infected swine herd.. J Virol.

[10] Dawood FS, Jain S, Finelli L, Shaw MW, Lindstrom, S (2009). Emergence of a novel swine-origin influenza A (H1N1) virus in humans.. N Engl J Med.

[11] Howden KJ, Brockhoff EJ, Caya FD, McLeod LJ, Lavoie M (2009). An investigation into human pandemic influenza virus (H1N1) 2009 on an Alberta swine farm.. Can Vet J.

[12] Mathieu C, Moreno V, Retama P, Gonzalez A, Rivera J (2010). Pandemic (H1N1) 2009 in breeding turkeys, Valparaiso, Chile.. Emerg Infect Dis.

[13] Berhane Y, Ojkic D, Neufeld J, Leith M, Hisanaga T (2010). Molecular characterization of pandemic H1N1 influenza viruses isolated from turkeys and pathogenicity of a human pH1N1 isolate in turkeys.. Avian Dis.

[14] Zhou NN, Senne DA, Landgraf JS, Swenson SL, Erickson G (1999). Genetic reassortment of avian, swine, and human influenza A viruses in American pigs.. J Virol.

[15] Reid AH, Fanning TG, Hultin JV, Taubenberger JK (1999). Origin and evolution of the 1918 "Spanish" influenza virus hemagglutinin gene.. Proc Natl Acad Sci USA.

[16] Webby RJ, Swenson SL, Krauss SL, Gerrish PJ, Goya SM (2000). Evolution of swine H3N2 influenza viruses in the United States.. J Virol.

[17] Vincent AL, Lager KM, Ma W, Lekcharoensuk P, Gramer MR (2006). Evaluation of hemagglutinin subtype 1 swine influenza viruses from the United States.. Vet Microbiol.

[18] Yassine HM, Lee CW, Suarez DL, Saif YM (2008). Genetic and antigenic relatedness of H3 subtype influenza A viruses isolated from avian and mammalian species.. Vaccine.

[19] Kapczynski DR, Gonder E, Liljebjelke K, Lippert R, Petkov D (2009). Vaccine-induced protection from egg production losses in commercial turkey breeder hens following experimental challenge with a triple-reassortant H3N2 avian influenza virus.. Avian Dis.

[20] Pillai SP, Pantin-Jackwood M, Jadhao SJ, Suarez DL, Wang L (2009). Pathobiology of triple reassortant H3N2 influenza viruses in breeder turkeys and its potential implication for vaccine studies in turkeys.. Vaccine.

[21] Young K, Lee JH, Erickson G, Goyal SM, Joo HS (2004). H3N2 influenza virus transmission to swine and turkeys, United States.. Emerg Infect Dis.

[22] Spackman E, Senne DA, Bulaga LL, Myers TJ, Perdue ML (2003). Development of real-time RT-PCR for the detection of avian influenza virus..

[23] Hoffmann E, Stech J, Guan Y, Webster RG, Perez DR (2001). Universal primer set for the full-length amplification of all influenza A viruses.. Arch Virol.

[24] Nfon C, Berhane Y, Zhang S, Handel H, Labrecque O (2011). Molecular and antigenic characterization of triple-reassortant H3N2 swine influenza viruses isolated from pigs, turkey and quail in Canada.. Transbound Emerg Dis.

[25] Ha Y, Stevens DJ, Skehel JJ, Wiley DC (2003). X-ray structure of the hemagglutinin of a potential H3 avian progenitor of the 1968 Hong Kong pandemic influenza virus.. Virology.

[26] Wiley DC, Wilson IA, Skehel JJ (1981). Structural identification of the antibody-binding sites of Hong Kong influenza haemagglutinin and their involvement in antigenic variation.. Nature.

[27] Weis W, Brown JH, Cusack S, Paulson JC, Skehel JJ (1988). Structure of the influenza virus haemagglutinin complexed with its receptor, sialic acid.. Nature.

[28] Tremblay D, Allard V, Doyon J-F, Bellehumeur C, Spearman G (2011). Emergence of a new swine H3N2 and pandemic (H1N1) 2009 influenza A virus in two Canadian animal populations, mink and swine.. J Clin Microbiol.

[29] Kumari K, Gulati S, Smith DF, Gulati U, Cummings RD (2007). Receptor binding specificity of recent human H3N2 influenza viruses.. Virology J.

[30] Nobusawa E, Ishihara H, Morishita T, Sato K, Nakajima K (2000). Change in receptor-binding specificity of recent human influenza A viruses (H3N2): a single amino acid change in hemagglutinin altered its recognition of sialyloligosaccharides.. Virology.

[31] Nakajima K, Nobusawa E, Tonegawa K, Nakajima S (2003). Restriction of amino acid change in influenza A virus H3HA: comparison of amino acid changes observed in nature and in vitro.. J Virol.

[32] Long J, Bushnell RV, Tobin JK, Pan K, Deem MW (2011). Evolution of H3N2 Influenza Virus in a Guinea Pig Model. PLoS One..

[33] Mishin VP, Novikov D, Hayden FG, Gubareva LV (2005). Effect of hemagglutinin glycosylation on influenza virus susceptibility to neuraminidase inhibitors.. J Virol.

[34] Vigerust DJ, Ulett KB, Boyd KL, Madsen J, Hawgood S (2007). N-linked glycosylation attenuates H3N2 influenza viruses.. J Virol.

[35] Wilson IA, Cox N (1990). Structural basis of immune recognition of influenza virus hemagglutinin.. Annu Rev Immunol.

[36] Igarashi M, Ito K, Kida H, Takada A (2008). Genetically destined potentials for N-linked glycosylation of influenza virus hemagglutinin.. Virology.

